# Genomic Analysis of a Community-Acquired Methicillin-Resistant *Staphylococcus aureus* Sequence Type 1 Associated with Caprine Mastitis

**DOI:** 10.3390/pathogens13010023

**Published:** 2023-12-26

**Authors:** Priscylla C. Vasconcelos, Elma L. Leite, Mauro M. S. Saraiva, Rafaela G. Ferrari, Samuel P. Cibulski, Nubia M. V. Silva, Oliveiro C. Freitas Neto, Patrícia E. N. Givisiez, Rafael F. C. Vieira, Celso J. B. Oliveira

**Affiliations:** 1 Department of Animal Science, College for Agricultural Sciences, Federal University of Paraiba (CCA/UFPB), Areia 58051-900, PB, Brazil; pczootecnista.1@gmail.com (P.C.V.); limaleiteelma@gmail.com (E.L.L.); mauro.saraiva@unesp.br (M.M.S.S.); rafaelaferrari@yahoo.com.br (R.G.F.); patricia.givisiez@academico.ufpb.br (P.E.N.G.); 2 School of Agricultural and Veterinarian Sciences, Department of Pathology, Reproduction, and One Health, São Paulo State University (Unesp), Jaboticabal 14884-900, SP, Brazil; 3 Center for Biotechnology (CBiotec), Federal University of Paraiba (CBiotec/UFPB), João Pessoa 58051-900, PB, Brazil; spcibulski@gmail.com; 4 Animal Production Center, National Institute of Semiarid (INSA), Campina Grande 58434-700, PB, Brazil; nubia274@gmail.com; 5 Department of Preventive Veterinary Medicine, Veterinary School, Federal University of Minas Gerais (UFMG), Belo Horizonte 31270-901, MG, Brazil; oliveirocaetano@yahoo.com.br; 6 Department of Public Health Sciences, The University of North Carolina at Charlotte, Charlotte, NC 28223, USA; 7 Center for Computational Intelligence to Predict Health and Environmental Risks (CIPHER), The University of North Carolina at Charlotte, Charlotte, NC 28223, USA

**Keywords:** antimicrobial resistance genes, goat milk, *mecA*, MRSA, plasmids, virulence genes, whole genome sequencing

## Abstract

This study aimed to investigate the genomic and epidemiological features of a methicillin-resistant *Staphylococcus aureus* sequence type 1 (MRSA ST1) strain associated with caprine subclinical mastitis. An *S. aureus* strain was isolated from goat’s milk with subclinical mastitis in Paraiba, Northeastern Brazil, by means of aseptic procedures and tested for antimicrobial susceptibility using the disk-diffusion method. Whole genome sequencing was performed using the Illumina MiSeq platform. After genome assembly and annotation, in silico analyses, including multilocus sequence typing (MLST), antimicrobial resistance and stress-response genes, virulence factors, and plasmids detection were performed. A comparative SNP-based phylogenetic analysis was performed using publicly available MRSA genomes. The strain showed phenotypic resistance to cefoxitin, penicillin, and tetracycline and was identified as sequence type 1 (ST1) and *spa* type 128 (t128). It harbored the SCC*mec* type IVa (2B), as well as the *luk*F-PV and *luk*S-PV genes. The strain was phylogenetically related to six community-acquired MRSA isolates (CA-MRSA) strains associated with human clinical disease in North America, Europe, and Australia. This is the first report of a CA-MRSA strain associated with milk in the Americas. The structural and epidemiologic features reported in the MRSA ST1 carrying a *mecA*-SCC*mec* type IVa suggest highly complex mechanisms of horizontal gene transfer in MRSA. The SNP-based phylogenetic analysis suggests a zooanthroponotic transmission, i.e., a strain of human origin.

## 1. Introduction

Although methicillin-resistant *Staphylococcus aureus* (MRSA) can be commonly found in the skin and nostrils of healthy individuals [1], an increasing number of infections associated with community-acquired and livestock-acquired MRSA strains have been reported in recent decades worldwide [2,3,4]. According to the World Health Organization [5], MRSA ranks as a high-priority organism for studies involving novel treatment options.

In dairy animals, MRSA-associated intra-mammary infections have been associated with frequent antimicrobial therapeutic failures, resulting in significant economic losses due to reduced production and compromised milk quality [6,7,8]. The tolerance to increased antimicrobial levels, especially β-lactams drugs, is related to the versatility of these bacteria in acquiring antimicrobial resistance and virulence genes by horizontal gene transfer mechanisms [2]. Therefore, mobile genetic elements (MGEs), such as genomic islands, bacteriophages, pathogenicity islands, insertion sequences, transposons, chromosomal cassettes, and plasmids, play a key role in the dissemination of resistance and virulence genes, contributing to the survival and plasticity of staphylococci [2,9,10]. Although antimicrobial resistance, virulence, and toxin-encoding genes contributing to the adaptation and rapid dissemination of staphylococci in different environments are commonly carried in plasmids [11,12], there is a lack of studies addressing this MGE in staphylococci.

Considering the global relevance of MRSA as a human pathogen and the emerging importance of animals and animal-derived products as sources of MRSA to humans, we reported herein an in-depth genomic investigation of a community-acquired methicillin-resistant *Staphylococcus aureus* (CA-MRSA) strain associated with subclinical caprine mastitis in Northeastern Brazil.

## 2. Materials and Methods

The *S. aureus* isolate, originally identified as SA31, was cultured from an udder half-milk sample aseptically collected from a lactating goat in Northeastern Brazil, according to the protocol of the National Mastitis Council [13]. We used a blood agar base (Oxoid, ThermoFisher, Waltham, MA, USA) supplemented with 5% defibrinated sheep blood for conventional microbiological isolation. The isolated was identified as *S. aureus* by means of Gram staining and biochemical testing, including catalase, oxidase, and tube coagulase. After confirmation by matrix-assisted laser desorption/ionization mass spectrometry (MALDI-TOF MS) (Bruker Daltonics, Bremen, Germany), the isolate was transferred into tryptic soy broth (TSB, BD), supplemented with 3.5 mg/L of cefoxitin, and incubated at 37 °C for 18 h. Then, 100 µL aliquot of inoculum was streaked onto mannitol salt agar (MSA, BD), also supplemented with cefoxitin (3.5 mg/L), and incubated at 37 °C for 18 h [14]. Antimicrobial susceptibility was determined using the disk-diffusion method according to the guidelines of the Clinical and Laboratory Standards Institute [15] using the following antibiotics and their respective concentrations: cefoxitin (FOX, 30 µg), ciprofloxacin (CIP, 5 µg), clindamycin (CLI, 2 µg), chloramphenicol (CHL-30 µg), erythromycin (ERY, 15 µg), gentamicin (GEN, 10 µg), oxacillin (OXA, 1 µg), penicillin (PEN, 10U), trimethoprim/sulfamethoxazole (SXT, 23.75/1.25 µg), and tetracycline (TET, 30 µg). *S. aureus* US400 reference strain was used as positive control. For storage, five copies of the isolate were kept at −70 °C in broth heart infusion (BHI, BD) supplemented with 40% glycerol.

Total DNA was extracted using a commercial extraction kit (Power Soil, Qiagen, Hilden, Germany) following the manufacturer’s protocol. After DNA integrity analysis in agarose gel and fluorometric quantification (Qubit 2.0, Life Technologies, Carlsbad, CA, USA), genomic libraries were prepared using the Nextera XT DNA Library Preparation kit (Illumina, San Diego, CA, USA). DNA fragment sizes were evaluated using a capillary electrophoresis system (Fragment Analyzer, Agilent, Waldbronn, Germany). Paired-end sequencing was performed in Illumina MiSeq using a v3 kit (2 × 150 cycles). The quality of the reads was checked using FastQC version 0.11.9 (https://www.bioinformatics.babraham.ac.uk/projects/fastqc/ accessed on 2 September 2023). The genome was de novo assembled using Unicycler [16]. Genomic-based predictions and functional annotations were performed using the PATRIC server [17].

The prediction of SA31 as a human pathogenic strain was performed by PathogenFinder 1.1 [18]. MLST 2.0 [19] was used for in silico multilocus sequence typing (MLST), and spaTyper 1.0 [20] for spa-typing was performed in silico using the Center for Genomic Epidemiology (CGE) server (https://cge.cbs.dtu.dk/services/ accessed on 3 September 2023). Virulence factors were screened using the Virulence Factors of Pathogenic Bacteria (VFDB) platform [21]. Antimicrobial resistance determinants were investigated using ResFinder 4.1 [22]. SCCmecFinder 1.2 [23] was used to identify the staphylococcal chromosomal cassette. Plasmids were detected by Plasmidfinder 2.1 [24] and manually curated in Geneious (v.9.0.5).

A comparative SNP-based phylogenetic analysis was performed using publicly available MRSA genomes using Isolates Browser (https://www.ncbi.nlm.nih.gov/pathogens/isolates accessed on 5 September 2023). AMRFinderPlus [25] was used for the detection of antimicrobial resistance determinants, stress response, and virulence genes.

## 3. Results and Discussion

*Staphylococcus aureus* SA31 was phenotypically resistant to β-lactams (FOX, OXA, PEN) and tetracycline (TET), as shown in Table 1 and Table S1 (Supplementary Materials).

**Figure 1:**
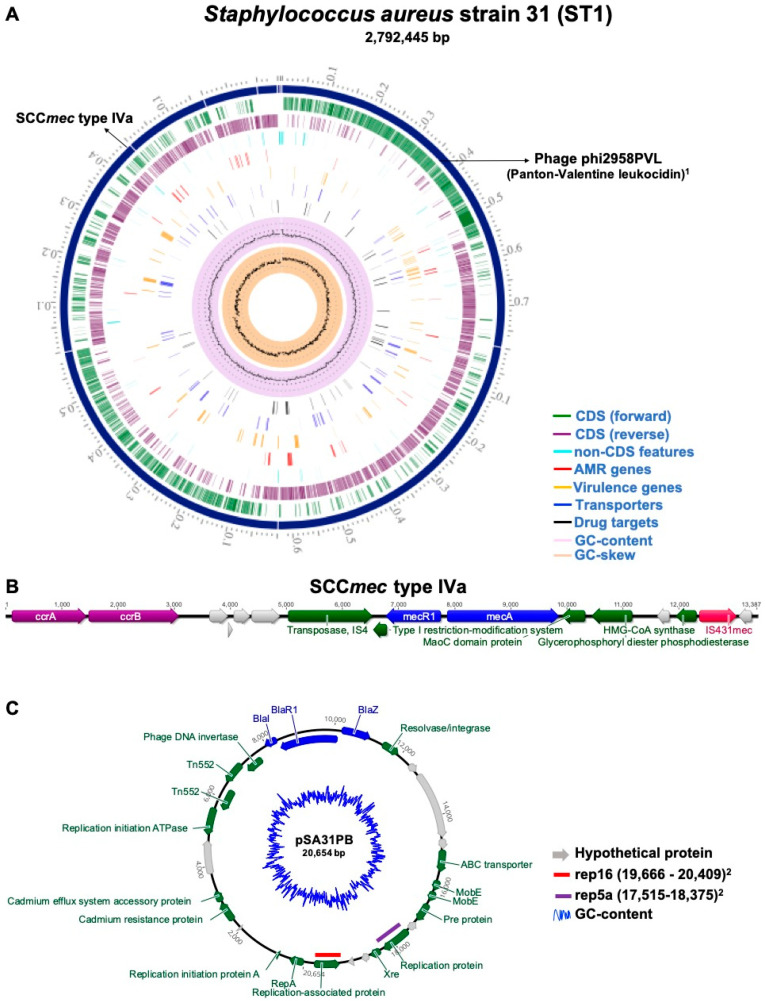
Circular genome map of the community-acquired *Staphylococcus aureus* strain 31 (CA-MRSA ST1) and genomic architecture of pSA31PB plasmid. (**A**) Genome map of CA-MRSA ST1 strain. From outer to inner ring: contigs (scale-x1Mbp), CDS on the forward strand, CDS on the reverse strand, RNA genes, CDS with homology to known antimicrobial resistance genes, CDS with homology to known virulence factors, GC content, and GC skew. The schematic figure was generated using the Comprehensive Genome Analysis Service in PATRIC server [17]. (**B**) Schematic representation of the SCC*mec* Type IVa showing the position of the *mec* complex, composed of IS431*mec*, *mecA,* and intact and truncated regions of the regulatory gene (*mecR1*), in addition to the *ccr* complex and the J regions, located between and around the *mec* and *ccr* complex. (**C**) Schematic representation of the 20,654 bp pSA31PB putative plasmid that harbors the *blaZ* gene and two genes replication *rep* types: *rep16* and *rep5a* Plasmid figure was generated using Geneious software (v. 9.0.5).

Sequencing generated 7,188,460 reads, averaging 118 bases and a total yield of 848,238,280 bases. A high-quality chromosome composed of contigs with an N50 of 628,879 and 303-fold coverage was assembled. The complete genome contained 2,792,445 bp, a G + C content of 32.69%, with a total of 2614 protein-coding sequences (CDS), 4 rRNA genes, and 57 tRNA genes. The graphical representation of the circular chromosome DNA is shown in Figure 1A.

In silico analyses revealed that the SA31 strain belonged to sequence type 1 (ST1) and *spa* type 128 (t128). It harbored a staphylococcal cassette chromosome *mec* element (SCC*mec*) type IVa (2B) carrying the *mecA* gene (Figure 1B). In addition, according to the pathogenicity prediction analysis, we observed a 97.6% probability of the strain being pathogenic to humans. SCC*mec* type IV carrying *mecA* antimicrobial resistance gene is commonly present in CA-MRSA strains [3,26,27,28]. Notably, skin and soft tissue infections caused by CA-MRSA sequence type 1 (ST1) strains carrying SCC*mec* IV have been reported in several countries [3,27,29]. The presence of SCC*mec* IVa in *S. aureus* associated with milk and dairy products [30,31,32,33] suggests a possible route of contamination by direct contact between dairy animals and humans during milking practices (including manual milking) or via the agricultural environment [32].

The MRSA ST1 strain 31 harbored the chromosomal *lmrS* gene, a multidrug efflux pump mechanism of the Major Facilitator Superfamily (MFS) conferring multidrug resistance in *S*. *aureus* strains, including chloramphenicol, erythromycin, and trimethoprim [34]. However, the investigated strain was phenotypically susceptible to these non-β-lactam antibiotics, similar to what has been reported in other MRSA ST1 strains [3,35,36,37]. Previous reports suggest that gene regulators capable of modulating the expression of multiple efflux pumps are involved in *lmrS* overexpression and, consequently, in the resistance phenotype [38,39]. According to Costa et al. [40], the upregulation of this gene is associated with exposure to sub-inhibitory concentrations of the antimicrobials.

The chromosomal sequence of the CA-MRSA ST1 strain harbored a set of virulence determinants, including *lukF-PV* and *lukS-PV* genes encoding the S and F subunits of Panton-Valentine leukocidin (PVL). Moreover, we identified a diversity of enterotoxin-encoding genes (*sea, seq, sek, sel, sec2,* and *seh*) and *icaC* gene involved in the externalization of the nascent polysaccharide [41], as well as the delta-hemolysin (*hld*), collagen adhesin (*cna*) and zinc metalloproteinase aureolysin (*aur*) genes (Table S2—Supplementary Materials). These genes were also identified by VFDB (Table 1).

Manual assembly of the putative plasmid generated a high-quality sequence with only one circular contig of 20,654 bp (pSA31PB). The annotation revealed 27 predicted open reading frames encoding proteins and a 28.37% G + C content. It harbors two encoding replication initiation (*rep*) genes: *rep16* (position 19666..20409; accession number: BX571858) and *rep5a* (position 17515..18375; accession number: AP003139). Detecting these rep-like sequences in plasmids is crucial as they are useful for staphylococcal plasmid classification [12,42]. In addition, these gene replication initiation sequences can be associated with antibiotic-resistance genes [12,43]. As shown in Figure 1C, pSA31PB co-harbored the stress response *cadD* gene and a set of antibiotic resistance genes, including the *blaZ*, associated with β-lactam resistance and usually found as part of the *bla* operon (*blaI, blaR1,* and *blaZ*) which is widely spread among Gram-positive bacteria [44]. The scarcity of characterization of plasmids in MRSA has been previously highlighted [11,12,43,45]. Considering the critical role of plasmids in the successful adaptation of strains, especially in those causing persistent MRSA infections [11,46,47], a better understanding of the genetic context of plasmids is extremely important to understand co-evolutionary events and the dissemination mechanisms of antimicrobial resistance.

**Figure 2:**
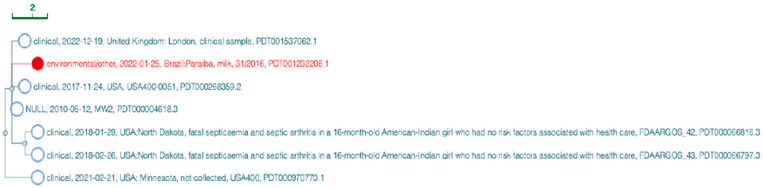
SNP-cluster tree generated by the NCBI Isolates Browser tool for the community-acquired methicillin-resistant *S. aureus* Sequence type 1 (CA-MRSA ST1 *S*) strain SA31 genome. The genome sequence of the CA-MRSA ST1 strain (SA31) causing intramammary subclinical infection in lactating goats in Northeastern Brazil is represented in red.

The phylogenetic position of SA31 compared to other CA-MRSA genomes performed by the NCBI Isolates Browser tool is shown in Figure 2. SA31 was highly related to six other MRSA strains of human clinical relevance, including a strain recently isolated in England (SAMN08815268) and the ST1-SCC*mec* IV MRSA strains USA400 (SAMN17703516) and USA400-0051 (SAMN05864218) in the USA. In Brazil, ST1-SCC*mec* IV isolates emerged as an important healthcare-associated pathogen [28,48,49]. Epidemiological data also demonstrate that CA-MRSA strains predominantly characterized as SCC*mec* IV have spread throughout the Brazilian territory [29,33,50]. Moreover, they are genetically related to highly virulent CA-MRSA MW2 strains (SAMN03255481; SAMN03255482; SAMD00061104) isolated in the 90s in the USA. The MW2 strain is one of the major pathogens causing community-acquired infections in the Midwestern USA. Several fatal infections were attributed to this strain in the late 1990s [51].

## 4. Conclusions

The presence of a CA-MRSA ST1 strain in goat milk, possibly transmitted by cross-contamination due to manual milking practices, indicates that milk and dairy products could eventually play a role in spreading these bacteria. The in-depth characterization of the CA-MRSA ST1 genome associated with mastitis in goat species, including the pSA31PB staphylococcal plasmid, can support future studies addressing the epidemiology, pathogenesis, and evolution of methicillin-resistant *Staphylococcus aureus*.

## Figures and Tables

**Table 1:** Genomic and phenotypic features of a community-associated methicillin-resistant *Staphylococcus aureus* (CA-MRSA) ST1 strain associated with subclinical caprine mastitis in Northeastern Brazil.

Features	*S. aureus* Strain 31
Phenotypic antibiotic resistance profile	FOX, OXA, PEN, TET
**Structural Genomic data**	
Genome size (bp)	2,792,445
Contigs number	9
CDSs	2614
rRNA	4
tRNA	57
% GC	32.69
**Epidemiological Genomic Data**	
MLST (ST) ^1^	ST1
SPATyper ^2^	t128
Plasmid ^3^	pSA31PB
ARG ^4,^*	*blaI*, *blaR1*, *blaZ*, *lmrS*, *mecA*, *tet(38)*
**Virulence factors (VFDB) ^5^**	
Adherence	*atl, ebh, clfA, clfB, cna, ebp, efb, fnbA, fnbB, icaA, icaB, icaC, icaR, sdrC, sdrD, sdrE, spa*
Enzyme	*sspA, sspB, sspC, hysA, geh, lip, splA, splB, splC, splF, coa, sak, nuc*
Immune evasion	*adsA, scn, sbi*
Secretion system	*esaA, esaB, esaD, esaE, esaG, essA, essB, essC, esxA, esxB, esxC, esxD*
Toxin	*hla, hld, sea, sec, seh, selk, sell, selq, set16, set17, set18, set19, set21, set22, set23, set24, set25, set26, set34, hlgA, hlgB, hlgC, lukD, lukF-PV, lukS-PV*

Legend: FOX—cefoxitin; OXA—oxacillin; PEN—penicillin; TET—tetracycline. * Antibiotic resistance genes. Available in ^1^ https://cge.cbs.dtu.dk/services/MLST/ (accessed on 5 September 2023), ^2^ https://cge.cbs.dtu.dk/services/spatyper/ (accessed on 5 September 2023), ^3^ https://cge.cbs.dtu.dk/services/PlasmidFinder/ (accessed on 5 September 2023); ^4^ https://cge.cbs.dtu.dk/services/ResFinder/ (accessed on 5 September 2023); ^5^ http://www.mgc.ac.cn/VFs/main.htm (accessed on 5 September 2023).

## Data Availability

Raw whole-genome sequencing data (Illumina pool-sequencing) have been deposited in the Sequence Read Archive under the accession number (SRR11665884), BioProject (PRJNA593524), BioSample (SAMN14792743). The Fasta file for the assemblies has been deposited in GenBank under the following accession code: JAHHIV000000000.

## Supplementary Materials

The following supporting information can be downloaded at https://www.mdpi.com/article/10.3390/pathogens13010023/s1. Table S1: Annotation data of the genome sequence of the CA-MRSA ST1 strain (SA31) causing intramammary subclinical infection in lactating goat in Northeastern Brazil. Table S2: Annotated resistance and virulence genes identified in the CA-MRSA ST1 strain (SA31) causing intramammary subclinical infection in lactating goat in Northeastern Brazil.
