# Supplementary material for: Genomic Analysis of a Community-Acquired Methicillin-Resistant Staphylococcus aureus Sequence Type 1 Associated with Caprine Mastitis
Source: Pathogens. 2023 Dec 26;13(1):23. doi: 10.3390/pathogens13010023 (PMC10819347; doi:10.3390/pathogens13010023)
Supplement: Supplementary file 1 [file pathogens-13-00023-s001.zip › pathogens-2727265-Supplementary Table S1.pdf]

Contig id	Start	Stop	Strand	Gene	Sequence name	Scope	Element type	Element subtype	Class	Subclass	Method	Target length	Ref. sequence length	% Coverage of ref. sequence	% Identity to reference sequence	Alignment length	Accession of closest sequence	Name of closest sequence
Staphylococcus_aureus_31_c1	38847	39779	+	lukE	bi-component leukocidin LukED subunit E	plus	VIRULENCE	VIRULENCE	NA	NA	BLASTX	311	311	100.00	99.68	311	RCV70079.1	bi-component leukocidin LukED subunit E
Staphylococcus_aureus_31_c1	39784	40764	+	lukD	bi-component leukocidin LukED subunit D	plus	VIRULENCE	VIRULENCE	NA	NA	BLASTX	327	327	100.00	94.50	327	CAA73668.1	bi-component leukocidin LukED subunit D
Staphylococcus_aureus_31_c1	52416	53120	+	splA	serine protease SplA	plus	VIRULENCE	VIRULENCE	NA	NA	BLASTX	235	235	100.00	99.57	235	ABD31004.1	serine protease SplA
Staphylococcus_aureus_31_c1	53248	53967	+	splB	serine protease SplB	plus	VIRULENCE	VIRULENCE	NA	NA	BLASTX	240	240	100.00	99.58	240	ABD31003.1	serine protease SplB
Staphylococcus_aureus_31_c1	427948	428883	+	lukS-PV	Panton-Valentine bi-component leukocidin subunit S	plus	VIRULENCE	VIRULENCE	NA	NA	BLASTX	312	312	100.00	99.68	312	AGW33874.1	Panton-Valentine bi-component leukocidin subunit S
Staphylococcus_aureus_31_c1	428888	429862	+	lukF-PV	Panton-Valentine bi-component leukocidin subunit F	plus	VIRULENCE	VIRULENCE	NA	NA	BLASTX	325	325	100.00	98.77	325	BAA24008.1	Panton-Valentine bi-component leukocidin subunit F
Staphylococcus_aureus_31_c2	321699	322418	+	sel	staphylococcal enterotoxin type L	plus	VIRULENCE	VIRULENCE	NA	NA	EXACTX	240	240	100.00	100.00	240	AMG40538.1	staphylococcal enterotoxin type L
Staphylococcus_aureus_31_c2	322591	323388	-	sec2	staphylococcal enterotoxin type C2	plus	VIRULENCE	VIRULENCE	NA	NA	BLASTX	266	266	100.00	99.25	266	AAA26624.1	staphylococcal enterotoxin type C2
Staphylococcus_aureus_31_c3	54324	57872	+	cna	collagen adhesin Cna	plus	VIRULENCE	VIRULENCE	NA	NA	BLASTX	1183	1183	100.00	98.99	1183	BAB96477.1	collagen adhesin Cna
Staphylococcus_aureus_31_c3	79565	80614	-	icaC	polysaccharide intercellular adhesin biosynthesis	plus	VIRULENCE	VIRULENCE	NA	NA	EXACTX	350	350	100.00	100.00	350	AUU58561.1	polysaccharide intercellular adhesin biosynthesis

					/export protein IcaC												/export protein IcaC
Staphylococcus_aureus_31_c3	124522	126048	+	aur	zinc metalloproteinase aureolysin	plus	VIRULENCE	VIRULENCE	NA	NA	BLASTX	509	509	100.00	99.41	509	EHO93748.1	zinc metalloproteinase aureolysin
Staphylococcus_aureus_31_c3	361426	362400	-	hlgB	bi-component gamma-hemolysin HlgAB/HlgCB subunit B	plus	VIRULENCE	VIRULENCE	NA	NA	BLASTX	325	325	100.00	97.54	325	CAA57278.1	bi-component gamma-hemolysin HlgAB/HlgCB subunit B
Staphylococcus_aureus_31_c3	362405	363349	-	hlgC	bi-component gamma-hemolysin HlgCB subunit C	plus	VIRULENCE	VIRULENCE	NA	NA	BLASTX	315	315	100.00	97.46	315	CAA57277.1	bi-component gamma-hemolysin HlgCB subunit C
Staphylococcus_aureus_31_c3	363920	364846	-	hlgA	bi-component gamma-hemolysin HlgAB subunit A	plus	VIRULENCE	VIRULENCE	NA	NA	EXACTX	309	309	100.00	100.00	309	AUU54669.1	bi-component gamma-hemolysin HlgAB subunit A
Staphylococcus_aureus_31_c3	597306	598745	+	lmrS	multidrug efflux MFS transporter LmrS	plus	STRESS	BIOCIDE	MACROLIDE/PHENICOL	CHLORAMPHENICOL/ERYTHROMYCIN	BLASTX	480	480	100.00	99.38	480	AAW38464.1	multidrug efflux MFS transporter LmrS
Staphylococcus_aureus_31_c4	129581	130933	-	mepA	multidrug efflux MATE transporter MepA	plus	AMR	AMR	EFFLUX	EFFLUX	BLASTX	451	451	100.00	99.33	451	BAB41547.1	multidrug efflux MATE transporter MepA
Staphylococcus_aureus_31_c4	360925	362274	-	tet(38)	tetracycline efflux MFS transporter Tet(38)	core	AMR	AMR	TETRACYCLINE	TETRACYCLINE	EXACTX	450	450	100.00	100.00	450	WP_001100294.1	tetracycline efflux MFS transporter Tet(38)
Staphylococcus_aureus_31_c4	429132	429854	-	seh	staphylococcal enterotoxin type H	plus	VIRULENCE	VIRULENCE	NA	NA	EXACTX	241	241	100.00	100.00	241	AAA19777.1	staphylococcal enterotoxin type H
Staphylococcus_aureus_31_c4	448421	449395	-	mecR1	beta-lactam sensor/signal transducer MecR1	core	AMR	AMR	BETA-LACTAM	METHICILLIN	PARTIALX	325	585	55.56	100.00	325	WP_000952923.1	beta-lactam sensor/signal transducer MecR1
Staphylococcus_aureus_31_c4	449495	451498	+	mecA	PBP2a family beta-	core	AMR	AMR	BETA-LACTAM	METHICILLIN	EXACTX	668	668	100.00	100.00	668	WP_000721310.1	PBP2a family beta-

					lactam-resistant peptidoglycan transpeptidase MecA													lactam-resistant peptidoglycan transpeptidase MecA
Staphylococcus_aureus_31_c5	83025	83372	-	scn	complement inhibitor SCIN-A	plus	VIRULENCE	VIRULENCE	NA	NA	EXACTX	116	116	100.00	100.00	116	ADL23798.1	complement inhibitor SCIN-A
Staphylococcus_aureus_31_c5	84873	85361	-	sak	staphylokinase	plus	VIRULENCE	VIRULENCE	NA	NA	BLASTX	163	163	100.00	99.39	163	CAA24957.1	staphylokinase
Staphylococcus_aureus_31_c5	87073	87843	-	sea	staphylococcal enterotoxin type A	plus	VIRULENCE	VIRULENCE	NA	NA	EXACTX	257	257	100.00	100.00	257	BAV60816.1	staphylococcal enterotoxin type A
Staphylococcus_aureus_31_c5	121762	122487	+	seq	staphylococcal enterotoxin type Q	plus	VIRULENCE	VIRULENCE	NA	NA	BLASTX	242	242	100.00	98.76	242	EYN56163.1	staphylococcal enterotoxin type Q
Staphylococcus_aureus_31_c5	122514	123236	+	sek	staphylococcal enterotoxin type K	plus	VIRULENCE	VIRULENCE	NA	NA	BLASTX	241	242	99.59	97.10	241	AAW36439.1	staphylococcal enterotoxin type K
Staphylococcus_aureus_31_c5	143296	143373	-	hld	delta-hemolysin	plus	VIRULENCE	VIRULENCE	NA	NA	BLASTX	26	26	100.00	96.15	26	AGU62139.1	delta-hemolysin
